# Directed Bee Colony Optimization Algorithm to Solve the Nurse Rostering Problem

**DOI:** 10.1155/2017/6563498

**Published:** 2017-04-04

**Authors:** M. Rajeswari, J. Amudhavel, Sujatha Pothula, P. Dhavachelvan

**Affiliations:** ^1^Department of CSE, Pondicherry University, Puducherry, India; ^2^Department of CSE, KL University, Andhra Pradesh, India

## Abstract

The Nurse Rostering Problem is an NP-hard combinatorial optimization, scheduling problem for assigning a set of nurses to shifts per day by considering both hard and soft constraints. A novel metaheuristic technique is required for solving Nurse Rostering Problem (NRP). This work proposes a metaheuristic technique called Directed Bee Colony Optimization Algorithm using the Modified Nelder-Mead Method for solving the NRP. To solve the NRP, the authors used a multiobjective mathematical programming model and proposed a methodology for the adaptation of a Multiobjective Directed Bee Colony Optimization (MODBCO). MODBCO is used successfully for solving the multiobjective problem of optimizing the scheduling problems. This MODBCO is an integration of deterministic local search, multiagent particle system environment, and honey bee decision-making process. The performance of the algorithm is assessed using the standard dataset INRC2010, and it reflects many real-world cases which vary in size and complexity. The experimental analysis uses statistical tools to show the uniqueness of the algorithm on assessment criteria.

## 1. Introduction

Metaheuristic techniques, especially the Bee Colony Optimization Algorithm, can be easily adapted to solve a larger number of NP-hard combinatorial optimization problems by combining other methods. The metaheuristic method can be divided into local search methods and global search methods. Local search methods such as tabu search, simulated annealing, and the Nelder-Mead Methods are used to exploit search space of the problem while global search methods such as scatter search, genetic algorithms, and Bee Colony Optimization focus on the exploration of the search space area [1]. Exploitation is the process of intensifying the search space; this method repeatedly restarts searching for each time from a different initial solution. Exploration is the process of diversifying the search space to evade trapping in a local optimum. A hybrid method is used to obtain a balance between exploration and exploitation by introducing local search within global search to obtain a robust solution for the NRP. In a previous study, the genetic algorithm was chosen for global search and simulated annealing for a local search to solve the NRP in [2].

In swarm intelligence, the natural behavior of organisms will follow a simple basic rule to structure their environment. The agents will not have any centralized structure to control other individuals; it uses the local interactions among the agents to determine the complex global behavior of the agents [3]. Some of the inspired natural behavior of swarm intelligence comprises bird flocking, ant colony, fish schooling, and animal herding methods. The various algorithms include the ant colony optimization algorithm, genetic algorithm, and the particle swarm optimization algorithm [4–6]. The natural foraging behavior of honey bees has inspired bee algorithm. All honey bees will start to collect nectar from various sites around their new hive, and the process of finding out the best nectar site is done by the group decision of honey bees. The mode of communication among the honey bees is carried out by the process of the waggle dance to inform hive mates about the location of rich food sources. Some of the algorithms which follow the waggle dance of communication performed by scout bees about the nectar site are bee system, Bee Colony Optimization [7], and Artificial Bee Colony [8].

The Directed Bee Colony (DBC) Optimization Algorithm [9] is inspired by the group decision-making process of bee behavior for the selection of the nectar site. The group decision process includes consensus and quorum methods. Consensus is the process of vote agreement, and the voting pattern of the scouts is monitored. The best nest site is selected once the quorum (threshold) value is reached. The experimental result shows that the algorithm is robust and accurate for generating the unique solution. The contribution of this research article is the use of a hybrid Directed Bee Colony Optimization with the Nelder-Mead Method for effective local search. The authors have adapted MODBCO for solving multiobjective problems which integrate the following processes: At first a deterministic local search method, Modified Nelder-Mead, is used to obtain the provisional optimal solution. Then a multiagent particle system environment is used in the exploration and decision-making process for establishing a new colony and nectar site selection. Only few honey bees were active in the process of decision-making, so the energy conservation of the swarm is highly achievable.

The Nurse Rostering Problem (NRP) is a staff scheduling problem that intends to assign a set of nurses to work shifts to maximize hospital benefit by considering a set of hard and soft constraints like allotment of duty hours, hospital regulations, and so forth, This nurse rostering is a delicate task of finding combinatorial solutions by satisfying multiple constraints [10]. Satisfying the hard constraint is mandatory in any scheduling problem, and a violation of any soft constraints is allowable but penalized. To achieve an optimal global solution for the problem is impossible in many cases [11]. Many algorithmic techniques such as metaheuristic method, graph-based heuristics, and mathematical programming model have been proposed to solve automated scheduling problems and timetabling problems over the last decades [12, 13].

In this work, the effectiveness of the hybrid algorithm is compared with different optimization algorithms using performance metrics such as error rate, convergence rate, best value, and standard deviation. The well-known combinatorial scheduling problem, NRP, is chosen as the test bed to experiment and analyze the effectiveness of the proposed algorithm.

This paper is organized as follows: Section 2 presents the literature survey of existing algorithms to solve the NRP.  Section 3 highlights the mathematical model and the formulation of hard and soft constraints of the NRP.  Section 4 explains the natural behavior of honey bees to handle decision-making process and the Modified Nelder-Mead Method.  Section 5 describes the development of the metaheuristic approach, and the effectiveness of the MODBCO algorithm to solve the NRP is demonstrated.  Section 6 confers the computational experiments and the analysis of results for the formulated problem. Finally, Section 7 provides the summary of the discussion and Section 8 will conclude with future directions of the research work.

## 2. Literature Review

Berrada et al. [19] considered multiple objectives to tackle the nurse scheduling problem by considering various ordered soft constraints. The soft constraints are ordered based on priority level, and this determines the quality of the solution. Burke et al. [20] proposed a multiobjective Pareto-based search technique and used simulated annealing based on a weighted-sum evaluation function towards preferences and a dominated-based evaluation function towards the Pareto set. Many mathematical models are proposed to reduce the cost and increase the performance of the task. The performance of the problem greatly depends on the type of constraints used [21]. Dowsland [22] proposed a technique of chain moves using a multistate tabu search algorithm. This algorithm exchanges the feasible and infeasible search space to increase the transmission rate when the system gets disconnected. But this algorithm fails to solve other problems in different search space instances.

Burke et al. [23] proposed a hybrid tabu search algorithm to solve the NRP in Belgian hospitals. In their constraints, the authors have added the previous roster along with hard and soft constraints. To consider this, they included heuristic search strategies in the general tabu search algorithm. This model provides flexibility and more user control. A hyperheuristic algorithm with tabu search is proposed for the NRP by Burke et al. [24]. They developed a rule based reinforcement learning, which is domain specific, but it chooses a little low-level heuristic to solve the NRP. The indirect genetic algorithm is problem dependent which uses encoding and decoding schemes with genetic operator to solve NRP. Burke et al. [25] developed a memetic algorithm to solve the nurse scheduling problem, and the authors have compared memetic and tabu search algorithm. The experimental result shows a memetic algorithm outperforms with better quality than the genetic algorithm and tabu search algorithm.

Simulated annealing has been proposed to solve the NRP. Hadwan and Ayob [26] introduced a shift pattern approach with simulated annealing. The authors have proposed a greedy constructive heuristic algorithm to generate the required shift patterns to solve the NRP at UKMMC (Universiti Kebangsaan Malaysia Medical Centre). This methodology will reduce the complexity of the search space solution to generate a roster by building two- or three-day shift patterns. The efficiency of this algorithm was shown by experimental results with respect to execution time, performance considerations, fairness, and the quality of the solution. This approach was capable of handling all hard and soft constraints and produces a quality roster pattern. Sharif et al. [27] proposed a hybridized heuristic approach with changes in the neighborhood descent search algorithm to solve the NRP at UKMMC. This heuristic is the hybridization of cyclic schedule with noncyclic schedule. They applied repairing mechanism, which swaps the shifts between nurses to tackle the random shift arrangement in the solution. A variable neighborhood descent search algorithm (VNDS) is used to change the neighborhood structure using a local search and generate a quality duty roster. In VNDS, the first neighborhood structure will reroster nurses to different shifts and the second neighborhood structure will do repairing mechanism.

Aickelin and Dowsland [28] proposed a technique for shift patterns; they considered shift patterns with penalty, preferences, and number of successive working days. The indirect genetic algorithm will generate various heuristic decoders for shift patterns to reconstruct the shift roster for the nurse. A qualified roster is generated using decoders with the help of the best permutations of nurses. To generate best search space solutions for the permutation of nurses, the authors used an adaptive iterative method to adjust the order of nurses as scheduled one by one. Asta et al. [29] and Anwar et al. [30] proposed a tensor-based hyperheuristic to solve the NRP. The authors tuned a specific group of datasets and embedded a tensor-based machine learning algorithm. A tensor-based hyperheuristic with memory management is used to generate the best solution. This approach is considered in life-long applications to extract knowledge and desired behavior throughout the run time.

Todorovic and Petrovic [31] proposed the Bee Colony Optimization approach to solve the NRP; all the unscheduled shifts are allocated to the available nurses in the constructive phase. This algorithm combines the constructive move with local search to improve the quality of the solution. For each forward pass, the predefined numbers of unscheduled shifts are allocated to the nurses and discarded the solution with less improvement in the objective function. The process of intelligent reduction in neighborhood search had improved the current solution. In construction phase, unassigned shifts are allotted to nurses and lead to violation of constraints to higher penalties.

Several methods have been proposed using the INRC2010 dataset to solve the NRP; the authors have considered five latest competitors to measure the effectiveness of the proposed algorithm. Asaju et al. [14] proposed Artificial Bee Colony (ABC) algorithm to solve NRP. This process is done in two phases; at first heuristic based ordering of shift pattern is used to generate the feasible solution. In the second phase, to obtain the solution, ABC algorithm is used. In this method, premature convergence takes place, and the solution gets trapped in local optima. The lack of a local search algorithm of this process leads to yielding higher penalty. Awadallah et al. [15] developed a metaheuristic technique hybrid artificial bee colony (HABC) to solve the NRP. In ABC algorithm, the employee bee phase was replaced by a hill climbing approach to increase exploitation process. Use of hill climbing in ABC generates a higher value which leads to high computational time.

The global best harmony search with pitch adjustment design is used to tackle the NRP in [16]. The author adapted the harmony search algorithm (HAS) in exploitation process and particle swarm optimization (PSO) in exploration process. In HAS, the solutions are generated based on three operator, namely, memory consideration, random consideration, and pitch adjustment for the improvisation process. They did two improvisations to solve the NRP, multipitch adjustment to improve exploitation process and replaced random selection with global best to increase convergence speed. The hybrid harmony search algorithm with hill climbing is used to solve the NRP in [17]. For local search, metaheuristic harmony and hill climbing approach are used. The memory consideration parameter in harmony is replaced by PSO algorithm. The derivative criteria will reduce the number of iterations towards local minima. This process considers many parameters to construct the roster since improvisation process is to be at each iteration.

Santos et al. [18] used integer programming (IP) to solve the NRP and proposed monolith compact IP with polynomial constraints and variables. The authors have used both upper and lower bounds for obtaining optimal cost. They estimated and improved lower bound values towards optimum, and this method requires additional processing time.

## 3. Mathematical Model

**Figure 1:**
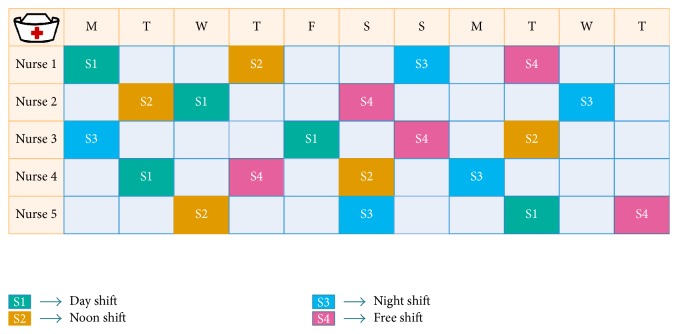
Illustrative example of Nurse Rostering Problem.

The NRP problem is a real-world problem at hospitals; the problem is to assign a predefined set of shifts (like S1-day shift, S2-noon shift, S3-night shift, and S4-Free-shift) of a scheduled period for a set of nurses of different preferences and skills in each ward.  Figure 1 shows the illustrative example of the feasible nurse roster, which consists of four shifts, namely, day shift, noon shift, night shift, and free shift (holiday), allocating five nurses over 11 days of scheduled period. Each column in the scheduled table represents the day and the cell content represents the shift type allocated to a nurse. Each nurse is allocated one shift per day and the number of shifts is assigned based on the hospital contracts. This problem will have some variants on a number of shift types, nurses, nurse skills, contracts, and scheduling period. In general, both hard and soft constraints are considered for generating and assessing solutions.

Hard constraints are the regulations which must be satisfied to achieve the feasible solution. They cannot be violated since hard constraints are demanded by hospital regulations. The hard constraints HC1 to HC5 must be filled to schedule the roster. The soft constraints SC1 to SC14 are desirable, and the selection of soft constraints determines the quality of the roster. Tables 1 and 2 list the set of hard and soft constraints considered to solve the NRP. This section describes the mathematical model required for hard and soft constraints extensively.

The NRP consists of a set of nurses *n* = 1,2,…, *N*, where each row is specific to particular set of shifts *s* = 1,2,…, *S*, for the given set day *d* = 1,2,…, *D*. The solution roster Ş for the 0/1 matrix dimension *N∗SD* is as in

(1) $$\begin{array}{c} {\text{Ş}}_{n,d,s}=\left\{\begin{array}{cc} 1 & \text{if nurse }n\text{ works }s\text{ shift for day }d\\ 0 & \text{otherwise.} \end{array}\right. \end{array}$$

*HC1*. In this constraint, all demanded shifts are assigned to a nurse.

(2) $$\begin{array}{c} \overset{N}{\underset{n=1}{\sum }}{\text{Ş}}_{d,s}^{n}=E_{ds},\;\forall d\in D,\;\;s\in S, \end{array}$$

where *E*_*ds*_ is the number of nurses required for a day (*d*) at shift (*s*) and Ş_*d*,*s*_ is the allocation of nurses in the feasible solution roster.

*HC2*. In this constraint, each nurse can work not more than one shift per day:

(3) $$\begin{array}{c} \overset{S}{\underset{s=1}{\sum }}{\text{Ş}}_{n,d}^{s}\leq 1,\;\forall n\in N,\;\;d\in D, \end{array}$$

where Ş_*n*,*d*_ is the allocation of nurses (*n*) in solution at shift (*s*) for a day (*d*).

*HC3*. This constraint deals with a minimum number of nurses required for each shift.

(4) $$\begin{array}{c} \overset{N}{\underset{n=1}{\sum }}{\text{Ş}}_{d,s}^{n}\geq {min}_{d,s}^{n},\;\forall d\in D,\;\;s\in S, \end{array}$$

where min_*d*,*s*_^*n*^ is the minimum number of nurses required for a shift (*s*) on the day (*d*).

*HC4*. In this constraint, the total number of working days for each nurse should range between minimum and maximum range for the given scheduled period.

(5) $$\begin{array}{c} W_{min}\leq \overset{D}{\underset{d=1}{\sum }}\;\overset{S}{\underset{s=1}{\sum }}{\text{Ş}}_{n}^{d,s}\leq W_{max},\;\forall n\in N. \end{array}$$

The average working shift for nurse can be determined by using

(6) $$\begin{array}{c} W_{avg}=\frac{1}{N}\left(\;\overset{D}{\underset{d=1}{\sum }}\;\overset{S}{\underset{s=1}{\sum }}{\text{Ş}}_{n}^{d,s},\;\;\forall n\in N\;\right), \end{array}$$

where *W*_min_ and *W*_max_ are the minimum and maximum number of days in scheduled period and *W*_avg_ is the average working shift of the nurse.

*HC5*. In this constraint, shift 1 followed by shift 3 is not allowed; that is, a day shift followed by a night shift is not allowed.

(7) $$\begin{array}{c} \overset{N}{\underset{n=1}{\sum }}\;\overset{D}{\underset{d=1}{\sum }}{\text{Ş}}_{s3}^{n,d}+{\text{Ş}}_{s1}^{n,d+1}\leq 1,\;\forall s\in S. \end{array}$$

*SC1*. The maximum number of shifts assigned to each nurse for the given scheduled period is as follows:

(8) $$\begin{array}{c} \max\left(\;\left(\;\overset{D}{\underset{d=1}{\sum }}\;\overset{S}{\underset{s=1}{\sum }}{\text{Ş}}_{n}^{d,s}-\Phi_{n}^{ub}\;\right),0\;\right),\;\forall n\in N, \end{array}$$

where Φ_*n*_^*ub*^ is the maximum number of shifts assigned to nurse (*n*).

*SC2*. The minimum number of shifts assigned to each nurse for the given scheduled period is as follows:

(9) $$\begin{array}{c} \max\left(\;\left(\;\Phi_{n}^{lb}-\overset{D}{\underset{d=1}{\sum }}\;\overset{S}{\underset{s=1}{\sum }}{\text{Ş}}_{n}^{d,s}\;\right),0\;\right),\;\forall n\in N, \end{array}$$

where Φ_*n*_^*lb*^ is the minimum number of shifts assigned to nurse (*n*).

*SC3*. The maximum number of consecutive working days assigned to each nurse on which a shift is allotted for the scheduled period is as follows:

(10) $$\begin{array}{c} \overset{\Psi_{n}}{\underset{k=1}{\sum }}\max\left(\;\left(\;C_{n}^{k}-\Theta_{n}^{ub}\;\right),0\;\right),\;\forall n\in N, \end{array}$$

where Θ_*n*_^*ub*^ is the maximum number of consecutive working days of nurse (*n*), Ψ_*n*_ is the total number of consecutive working spans of nurse (*n*) in the roster, and *ℂ*_*n*_^*k*^ is the count of the *k*th working spans of nurse (*n*).

*SC4*. The minimum number of consecutive working days assigned to each nurse on which a shift is allotted for the scheduled period is as follows:

(11) $$\begin{array}{c} \overset{\Psi_{n}}{\underset{k=1}{\sum }}\max\left(\;\left(\;{\Theta_{n}^{lb}-C}_{n}^{k}\;\right),0\;\right),\;\forall n\in N, \end{array}$$

where Θ_*n*_^*lb*^ is the minimum number of consecutive working days of nurse (*n*), Ψ_*n*_ is the total number of consecutive working spans of nurse (*n*) in the roster, and *ℂ*_*n*_^*k*^ is the count of the *k*th working span of the nurse (*n*).

*SC5*. The maximum number of consecutive working days assigned to each nurse on which no shift is allotted for the given scheduled period is as follows:

(12) $$\begin{array}{c} \overset{\Gamma_{n}}{\underset{k=1}{\sum }}\max\left(\;\left(\;{ð}_{n}^{k}-\varphi_{n}^{ub}\;\right),0\;\right),\;\forall n\in N, \end{array}$$

where *φ*_*n*_^*ub*^ is the maximum number of consecutive free days of nurse (*n*), Γ_*n*_ is the total number of consecutive free working spans of nurse (*n*) in the roster, and *ð*_*n*_^*k*^ is the count of the *k*th working span of the nurse (*n*).

*SC6*. The minimum number of consecutive working days assigned to each nurse on which no shift is allotted for the given scheduled period is as follows:

(13) $$\begin{array}{c} \overset{\Gamma_{n}}{\underset{k=1}{\sum }}\max\left(\;\left(\;\varphi_{n}^{lb}-{ð}_{n}^{k}\;\right),0\;\right),\;\forall n\in N, \end{array}$$

where *φ*_*n*_^*lb*^ is the minimum number of consecutive free days of nurse (*n*), Γ_*n*_ is the total number of consecutive free working spans of nurse (*n*) in the roster, and *ð*_*n*_^*k*^ is the count of the *k*th working span of the nurse (*n*).

*SC7*. The maximum number of consecutive working weekends with at least one shift assigned to nurse for the given scheduled period is as follows:

(14) $$\begin{array}{c} \overset{{\ddot{\Upsilon }}_{n}}{\underset{k=1}{\sum }}\max\left(\;\left(\;\zeta_{n}^{k}-\Omega_{n}^{ub}\;\right),0\;\right),\;\forall n\in N, \end{array}$$

where *Ω*_*n*_^*ub*^ is the maximum number of consecutive working weekends of nurse (*n*), ${\ddot{\Upsilon }}_{n}$ is the total number of consecutive working weekend spans of nurse (*n*) in the roster, and *ζ*_*n*_^*k*^ is the count of the *k*th working weekend span of the nurse (*n*).

*SC8*. The minimum number of consecutive working weekends with at least one shift assigned to nurse for the given scheduled period is as follows:

(15) $$\begin{array}{c} \overset{{\ddot{\Upsilon }}_{n}}{\underset{k=1}{\sum }}\max\left(\;\left(\;{\Omega_{n}^{lb}-\zeta }_{n}^{k}\;\right),0\;\right),\;\forall n\in N, \end{array}$$

where *Ω*_*n*_^*lb*^ is the minimum number of consecutive working weekends of nurse (*n*), ${\ddot{\Upsilon }}_{n}$ is the total number of consecutive working weekend spans of nurse (*n*) in the roster, and *ζ*_*n*_^*k*^ is the count of the *k*th working weekend span of the nurse (*n*).

*SC9*. The maximum number of weekends with at least one shift assigned to nurse in four weeks is as follows:

(16) $$\begin{array}{c} \overset{{\ddot{I}}_{n}}{\underset{k=1}{\sum }}\max\left(\;\left(\;{ϣ}_{n}^{k}-\varpi_{n}^{ub}\;\right),0\;\right),\;\forall n\in N, \end{array}$$

where *ϣ*_*n*_^*k*^ is the number of working days at the *k*th weekend of nurse (*n*), *ϖ*_*n*_^*ub*^ is the maximum number of working days for nurse (*n*), and ${\ddot{I}}_{n}$ is the total count of the weekend in the scheduling period of nurse (*n*).

*SC10*. The nurse can request working on a particular day for the given scheduled period.

(17) $$\begin{array}{c} \overset{D}{\underset{d=1}{\sum }}\lambda_{n}^{d}=1,\;\forall n\in N, \end{array}$$

where *λ*_*n*_^*d*^ is the day request from the nurse (*n*) to work on any shift on a particular day (*d*).

*SC11*. The nurse can request that they do not work on a particular day for the given scheduled period.

(18) $$\begin{array}{c} \overset{D}{\underset{d=1}{\sum }}\lambda_{n}^{d}=0,\;\forall n\in N, \end{array}$$

where *λ*_*n*_^*d*^ is the request from the nurse (*n*) not to work on any shift on a particular day (*d*).

*SC12*. The nurse can request working on a particular shift on a particular day for the given scheduled period.

(19) $$\begin{array}{c} \overset{D}{\underset{d=1}{\sum }}\;\overset{S}{\underset{s=1}{\sum }}{ϒ}_{n}^{d,s}=1,\;\forall n\in N, \end{array}$$

where *ϒ*_*n*_^*d*,*s*^ is the shift request from the nurse (*n*) to work on a particular shift (*s*) on particular day (*d*).

*SC13*. The nurse can request that they do not work on a particular shift on a particular day for the given scheduled period.

(20) $$\begin{array}{c} \overset{D}{\underset{d=1}{\sum }}\;\overset{S}{\underset{s=1}{\sum }}{ϒ}_{n}^{d,s}=0,\;\forall n\in N, \end{array}$$

where *ϒ*_*n*_^*d*,*s*^ is the shift request from the nurse (*n*) not to work on a particular shift (*s*) on particular day (*d*).

*SC14*. The nurse should not work on unwanted pattern suggested for the scheduled period.

(21) $$\begin{array}{c} \overset{\varrho_{n}}{\underset{u=1}{\sum }}\mu_{n}^{u},\;\forall n\in N, \end{array}$$

where *μ*_*n*_^*u*^ is the total count of occurring patterns for nurse (*n*) of type *u*; *ϱ*_*n*_ is the set of unwanted patterns suggested for the nurse (*n*).

The objective function of the NRP is to maximize the nurse preferences and minimize the penalty cost from violations of soft constraints in (22).

(22) min⁡fŞn,d,s=∑SC=114Psc∑n=1N ∑s=1S ∑d=1DŞn,d,s∗Tsc∑n=1N ∑s=1S ∑d=1DŞn,d,s.

Here SC refers to the set of soft constraints indexed in Table 2, *P*_sc_(*x*) refers to the penalty weight violation of the soft constraint, and *T*_sc_(*x*) refers to the total violations of the soft constraints in roster solution. It has to be noted that the usage of penalty function [32] in the NRP is to improve the performance and provide the fair comparison with another optimization algorithm.

## 4. Bee Colony Optimization

### 4.1. Natural Behavior of Honey Bees

Swarm intelligence is an emerging discipline for the study of problems which requires an optimal approach rather than the traditional approach. The use of swarm intelligence is the part of artificial intelligence based on the study of the behavior of social insects. The swarm intelligence is composed of many individual actions using decentralized and self-organized system. Swarm behavior is characterized by natural behavior of many species such as fish schools, herds of animals, and flocks of birds formed for the biological requirements to stay together. Swarm implies the aggregation of animals such as birds, fishes, ants, and bees based on the collective behavior. The individual agents in the swarm will have a stochastic behavior which depends on the local perception of the neighborhood. The communication between any insects can be formed with the help of the colonies, and it promotes collective intelligence among the colonies.

The important features of swarms are proximity, quality, response variability, stability, and adaptability. The proximity of the swarm must be capable of providing simple space and time computations, and it should respond to the quality factors. The swarm should allow diverse activities and should not be restricted among narrow channels. The swarm should maintain the stability nature and should not fluctuate based on the behavior. The adaptability of the swarm must be able to change the behavior mode when required. Several hundreds of bees from the swarm work together to find nesting sites and select the best nest site. Bee Colony Optimization is inspired by the natural behavior of bees. The bee optimization algorithm is inspired by group decision-making processes of honey bees. A honey bee searches the best nest site by considering speed and accuracy.

In a bee colony there are three different types of bees, a single queen bee, thousands of male drone bees, and thousands of worker bees.

1. The queen bee is responsible for creating new colonies by laying eggs.
2. The male drone bees mated with the queen and were discarded from the colonies.
3. The remaining female bees in the hive are called worker bees, and they are called the building block of the hive. The responsibilities of the worker bees are to feed, guard, and maintain the honey bee comb.

Based on the responsibility, worker bees are classified as scout bees and forager bees. A scout bee flies in search of food sources randomly and returns when the energy gets exhausted. After reaching a hive scout bees share the information and start to explore rich food source locations with forager bees. The scout bee's information includes direction, quality, quantity, and distance of the food source they found. The way of communicating information about a food source to foragers is done using dance. There are two types of dance, round dance and waggle dance. The round dance will provide direction of the food source when the distance is small. The waggle dance indicates the position and the direction of the food source; the distance can be measured by the speed of the dance. A greater speed indicates a smaller distance; and the quantity of the food depends on the wriggling of the bee. The exchange of information among hive mates is to acquire collective knowledge. Forager bees will silently observe the behavior of scout bee to acquire knowledge about the directions and information of the food source.

The group decision process of honey bees is for searching best food source and nest site. The decision-making process is based on the swarming process of the honey bee. Swarming is the process in which the queen bee and half of the worker bees will leave their hive to explore a new colony. The remaining worker bees and daughter bee will remain in the old hive to monitor the waggle dance. After leaving their parental hive, swarm bees will form a cluster in search of the new nest site. The waggle dance is used to communicate with quiescent bees, which are inactive in the colony. This provides precise information about the direction of the flower patch based on its quality and energy level. The number of follower bees increases based on the quality of the food source and allows the colony to gather food quickly and efficiently. The decision-making process can be done in two methods by swarm bees to find the best nest site. They are consensus and quorum; consensus is the group agreement taken into account and quorum is the decision process taken when the bee vote reaches a threshold value.

**Figure 2:**
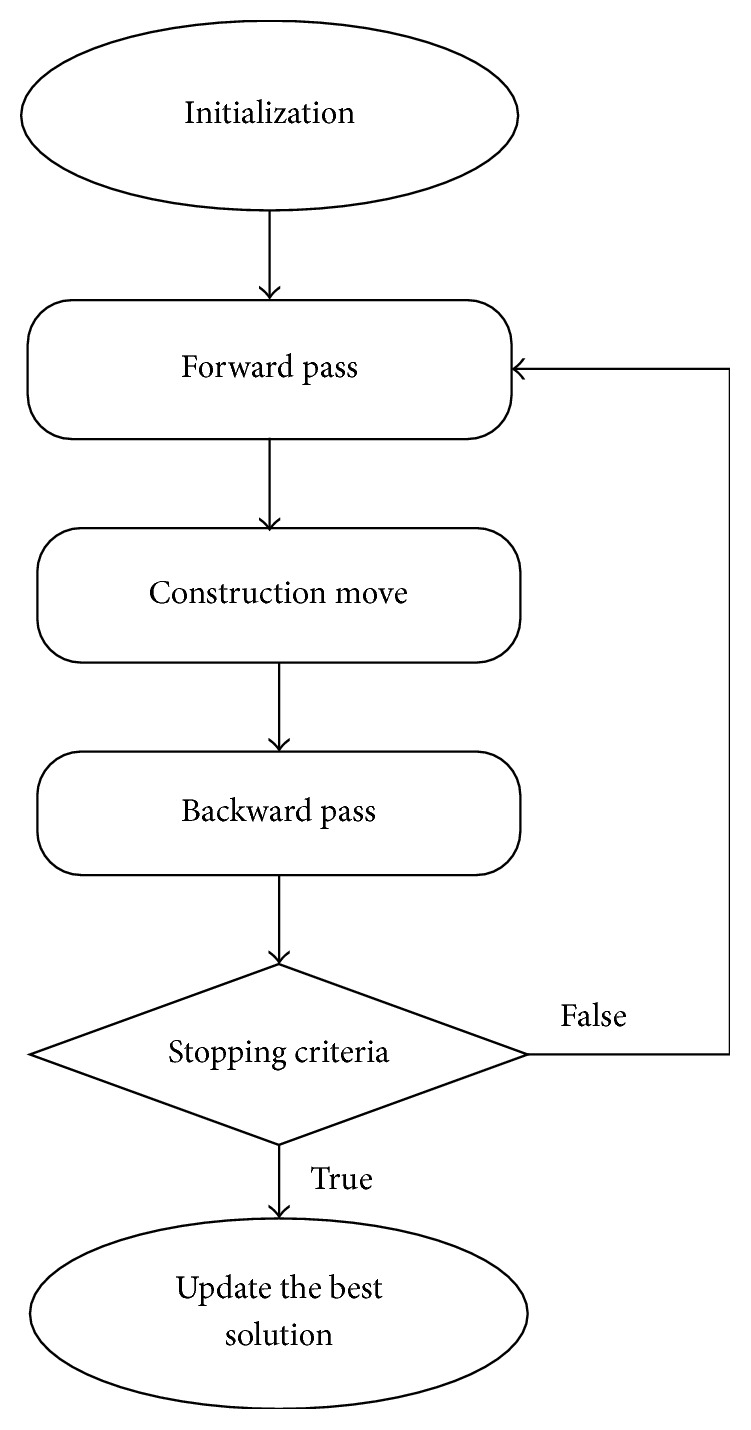
Flowchart of BCO algorithm.

Bee Colony Optimization (BCO) algorithm is a population-based algorithm. The bees in the population are artificial bees, and each bee finds its neighboring solution from the current path. This algorithm has a forward and backward process. In forwarding pass, every bee starts to explore the neighborhood of its current solution and enables constructive and improving moves. In forward pass, entire bees in the hive will start the constructive move and then local search will start. In backward pass, bees share the objective value obtained in the forward pass. The bees with higher priority are used to discard all nonimproving moves. The bees will continue to explore in next forward pass or continue the same process with neighborhood. The flowchart for BCO is shown in Figure 2. The BCO is proficient in solving combinatorial optimization problems by creating colonies of the multiagent system. The pseudocode for BCO is described in Algorithm 1. The bee colony system provides a standard well-organized and well-coordinated teamwork, multitasking performance [33].

### 4.2. Modified Nelder-Mead Method

The Nelder-Mead Method is a simplex method for finding a local minimum function of various variables and is a local search algorithm for unconstrained optimization problems. The whole search area is divided into different fragments and filled with bee agents. To obtain the best solution, each fragment can be searched by its bee agents through Modified Nelder-Mead Method (MNMM). Each agent in the fragments passes information about the optimized point using MNMM. By using NMMM, the best points are obtained, and the best solution is chosen by decision-making process of honey bees. The algorithm is a simplex-based method, *D*-dimensional simplex is initialized with *D* + 1 vertices, that is, two dimensions, and it forms a triangle; if it has three dimensions, it forms a tetrahedron. To assign the best and worst point, the vertices are evaluated and ordered based on the objective function.

**Figure 3:**
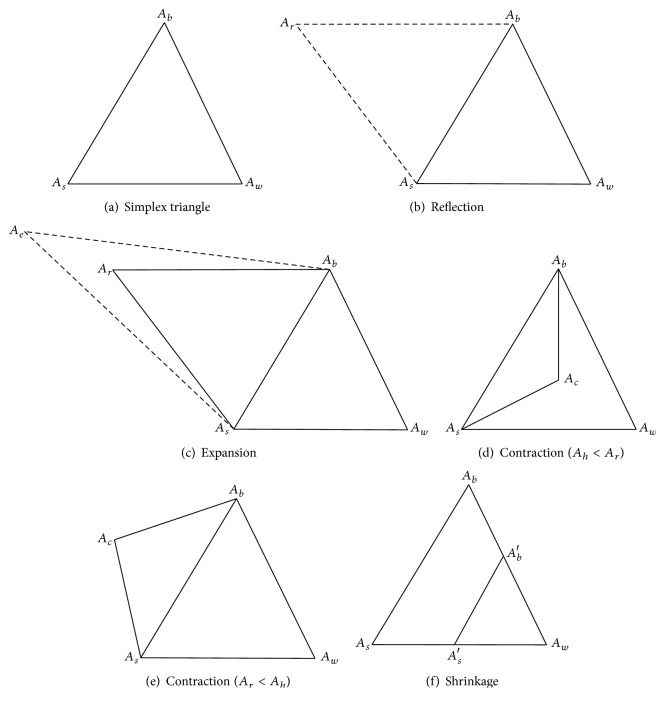
Nelder-Mead operations.

The best point or vertex is considered to the minimum value of the objective function, and the worst point is chosen with a maximum value of the computed objective function. To form simplex new vertex function values are computed. This method can be calculated using four procedures, namely, reflection, expansion, contraction, and shrinkage.  Figure 3 shows the operators of the simplex triangle in MNMM.

The simplex operations in each vertex are updated closer to its optimal solution; the vertices are ordered based on fitness value and ordered. The best vertex is *A*_*b*_, the second best vertex is *A*_*s*_, and the worst vertex is *A*_*w*_ calculated based on the objective function. Let *A* = (*x*, *y*) be the vertex in a triangle as food source points; *A*_*b*_ = (*x*_*b*_, *y*_*b*_), *A*_*s*_ = (*x*_*s*_, *y*_*s*_) and *A*_*w*_ = (*x*_*w*_, *y*_*w*_) are the positions of the food source points, that is, local optimal points. The objective functions for *A*_*b*_, *A*_*s*_, and *A*_*w*_ are calculated based on (23) towards the food source points.

The objective function to construct simplex to obtain local search using MNMM is formulated as

(23) $$\begin{array}{c} f\left(\;x,y\;\right)=x^{2}-4x+y^{2}-y-xy. \end{array}$$

**Figure 4:**
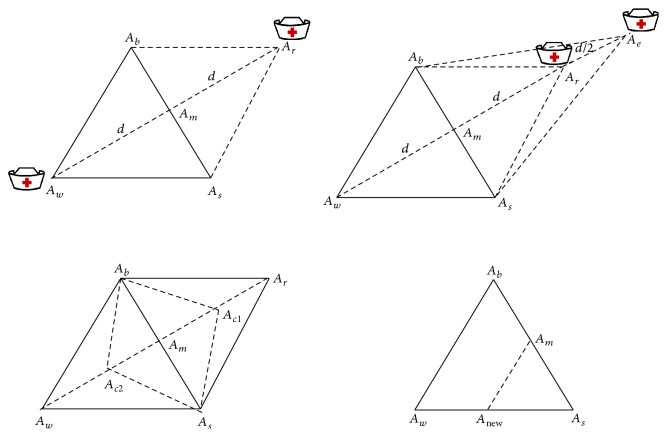
Bees search movement based on MNMM.

Based on the objective function value the vertices food points are ordered ascending with their corresponding honey bee agents. The obtained values are ordered as *A*_*b*_ ≤ *A*_*s*_ ≤ *A*_*w*_ with their honey bee position and food points in the simplex triangle.  Figure 4 describes the search of best-minimized cost value for the nurse based on objective function (22). The working principle of Modified Nelder-Mead Method (MNMM) for searching food particles is explained in detail.

(1) In the simplex triangle the reflection coefficient *α*, expansion coefficient *γ*, contraction coefficient *β*, and shrinkage coefficient *δ* are initialized.
(2) The objective function for the simplex triangle vertices is calculated and ordered. The best vertex with lower objective value is *A*_*b*_, the second best vertex is *A*_*s*_, and the worst vertex is named as *A*_*w*_, and these vertices are ordered based on the objective function as *A*_*b*_ ≤ *A*_*s*_ ≤ *A*_*w*_.
(3) The first two best vertices are selected, namely, *A*_*b*_ and *A*_*s*_, and the construction proceeds with calculating the midpoint of the line segment which joins the two best vertices, that is, food positions. The objective function decreases as the honey agent associated with the worst position vertex moves towards best and second best vertices. The value decreases as the honey agent moves towards *A*_*w*_ to *A*_*b*_ and *A*_*w*_ to *A*_*s*_. It is feasible to calculate the midpoint vertex *A*_*m*_ by the line joining best and second best vertices using
  (24) $$\begin{array}{c} A_{m}=\frac{A_{b}+A_{s}}{2}. \end{array}$$
(4) A reflecting vertex *A*_*r*_ is generated by choosing the reflection of worst point *A*_*w*_. The objective function value for *A*_*r*_ is *f*(*A*_*r*_) which is calculated, and it is compared with worst vertex *A*_*w*_ objective function value *f*(*A*_*w*_). If *f*(*A*_*r*_) < *f*(*A*_*w*_) proceed with step (5), the reflection vertex can be calculated using
  (25) $$\begin{array}{c} A_{r}=A_{m}+\alpha \left(\;A_{m}-A_{w}\;\right),\;\text{where}\;\;\alpha >0. \end{array}$$
(5) The expansion process starts when the objective function value at reflection vertex *A*_*r*_ is lesser than worst vertex *A*_*w*_, *f*(*A*_*r*_) < *f*(*A*_*w*_), and the line segment is further extended to *A*_*e*_ through *A*_*r*_ and *A*_*w*_. The vertex point *A*_*e*_ is calculated by (26). If the objective function value at *A*_*e*_ is lesser than reflection vertex *A*_*r*_, *f*(*A*_*e*_) < *f*(*A*_*r*_), then the expansion is accepted, and the honey bee agent has found best food position compared with reflection point.
  (26) $$\begin{array}{c} A_{e}=A_{r}+\gamma \left(\;A_{r}-A_{m}\;\right),\;\text{where}\;\;\gamma >1. \end{array}$$
(6) The contraction process is carried out when *f*(*A*_*r*_) < *f*(*A*_*s*_) and *f*(*A*_*r*_) ≤ *f*(*A*_*b*_) for replacing *A*_*b*_ with *A*_*r*_. If *f*(*A*_*r*_) > *f*(*A*_*h*_) then the direct contraction without the replacement of *A*_*b*_ with *A*_*r*_ is performed. The contraction vertex *A*_*c*_ can be calculated using
  (27) $$\begin{array}{c} A_{c}=\beta A_{r}+\left(\;1-\beta \;\right)A_{m},\;\text{where}\;\;0<\beta <1. \end{array}$$
If *f*(*A*_*r*_) ≤ *f*(*A*_*b*_), the contraction can be done and *A*_*c*_ replaced with *A*_*h*_; go to step (8) or else proceed to step (7).
(7) The shrinkage phase proceeds when the contraction process at step (6) fails and is done by shrinking all the vertices of the simplex triangle except *A*_*h*_ using (28). The objective function value of reflection and contraction phase is not lesser than the worst point; then the vertices *A*_*s*_ and *A*_*w*_ must be shrunk towards *A*_*h*_. Thus the vertices of smaller value will form a new simplex triangle with another two best vertices.
  (28) $$\begin{array}{c} A_{i}=\delta A_{i}+A_{1}\left(\;1-\delta \;\right),\;\text{where}\;\;0<\delta <1. \end{array}$$
(8) The calculations are stopped when the termination condition is met.

**Figure 5:**
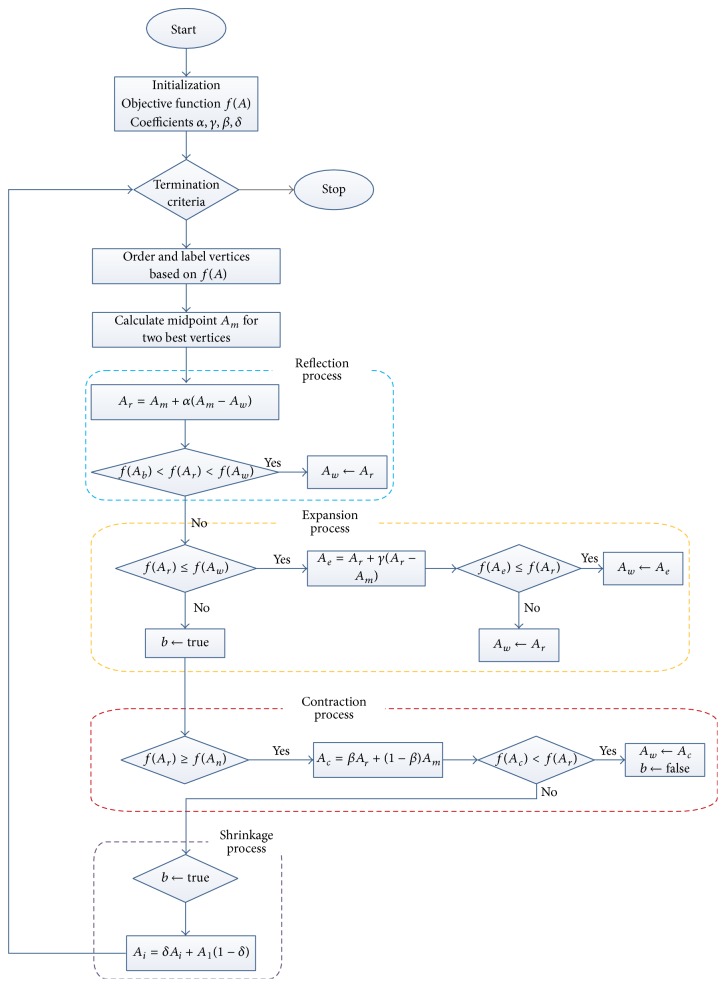
Workflow of Modified Nelder-Mead Method.

Algorithm 2 describes the pseudocode for Modified Nelder-Mead Method in detail. It portraits the detailed process of MNMM to obtain the best solution for the NRP. The workflow of the proposed MNMM is explained in Figure 5.

## 5. MODBCO

Bee Colony Optimization is the metaheuristic algorithm to solve various combinatorial optimization problems, and it is inspired by the natural behavior of bee for their food sources. The algorithm consists of two steps, forward and backward pass. During forwarding pass, bees started to explore the neighborhood of its current solution and find all possible ways. In backward pass, bees return to the hive and share the values of the objective function of their current solution. Calculate nectar amount using probability function and advertise the solution; the bee which has the better solution is given higher priority. The remaining bees based on the probability value decide whether to explore the solution or proceed with the advertised solution. Directed Bee Colony Optimization is the computational system where several bees work together in uniting and interact with each other to achieve goals based on the group decision process. The whole search area of the bee is divided into multiple fragments; different bees are sent to different fragments. The best solution in each fragment is obtained by using a local search algorithm Modified Nelder-Mead Method (MNMM). To obtain the best solution, the total varieties of individual parameters are partitioned into individual volumes. Each volume determines the starting point of the exploration of food particle by each bee. The bees use developed MNMM algorithm to find the best solution by remembering the last two best food sites they obtained. After obtaining the current solution, the bee starts to backward pass, sharing of information obtained during forwarding pass. The bees started to share information about optimized point by the natural behavior of bees called waggle dance. When all the information about the best food is shared, the best among the optimized point is chosen using a decision-making process called consensus and quorum method in honey bees [34, 35].

### 5.1. Multiagent System

All agents live in an environment which is well structured and organized. In multiagent system, several agents work together and interact with each other to obtain the goal. According to Jiao and Shi [36] and Zhong et al. [37] all agents should possess the following qualities: agents should live and act in an environment, each agent should sense its local environment, each agent should be capable of interacting with other agents in a local environment, and agents attempt to perform their goal. All agents interact with each other and take the decision to achieve the desired goals. The multiagent system is a computational system and provides an opportunity to optimize and compute all complex problems. In multiagent system, all agents start to live and act in the same environment which is well organized and structured. Each agent in the environment is fixed on a lattice point. The size and dimension of the lattice point in the environment depend upon the variables used. The objective function can be calculated based on the parameters fixed.

(1) Consider “*e*” number of independent parameters to calculate the objective function. The range of the *g*th parameter can be calculated using [*Q*_*gi*_, *Q*_*gf*_], where *Q*_*gi*_ is the initial value of the *g*th parameter and *Q*_*gf*_ is the final value of the *g*th parameter chosen.
(2) Thus the objective function can be formulated as *e* number of axes; each axis will contain a total range of single parameter with different dimensions.
(3) Each axis is divided into smaller parts; each part is called a step. So *g*th axis can be divided into *n*_*g*_ number of steps each with the length of *L*_*g*_, where the value of *g* depends upon parameters; thus *g* = 1 to *e*. The relationship between *n*_*g*_ and *L*_*g*_ can be given as
  (29) $$\begin{array}{c} n_{g}=\frac{Q_{gi}-Q_{gf}}{L_{g}}. \end{array}$$
(4) Then each axis is divided into branches, for each branch *g* number of branches will form an *e-*dimensional volume. Total number of volumes *N*_*v*_ can be formulated using
  (30) $$\begin{array}{c} N_{v}=\overset{e}{\underset{g=1}{\prod }}n_{g}. \end{array}$$
(5) The starting point of the agent in the environment, which is one point inside volume, is chosen by calculating the midpoint of the volume. The midpoint of the lattice can be calculated as
  (31) $$\begin{array}{c} \left[\;\frac{Q_{i1}-Q_{f1}}{2},\frac{Q_{i2}-Q_{f2}}{2},\ldots ,\frac{Q_{ie}-Q_{fe}}{2}\;\right]. \end{array}$$

### 5.2. Decision-Making Process

**Figure 6:**
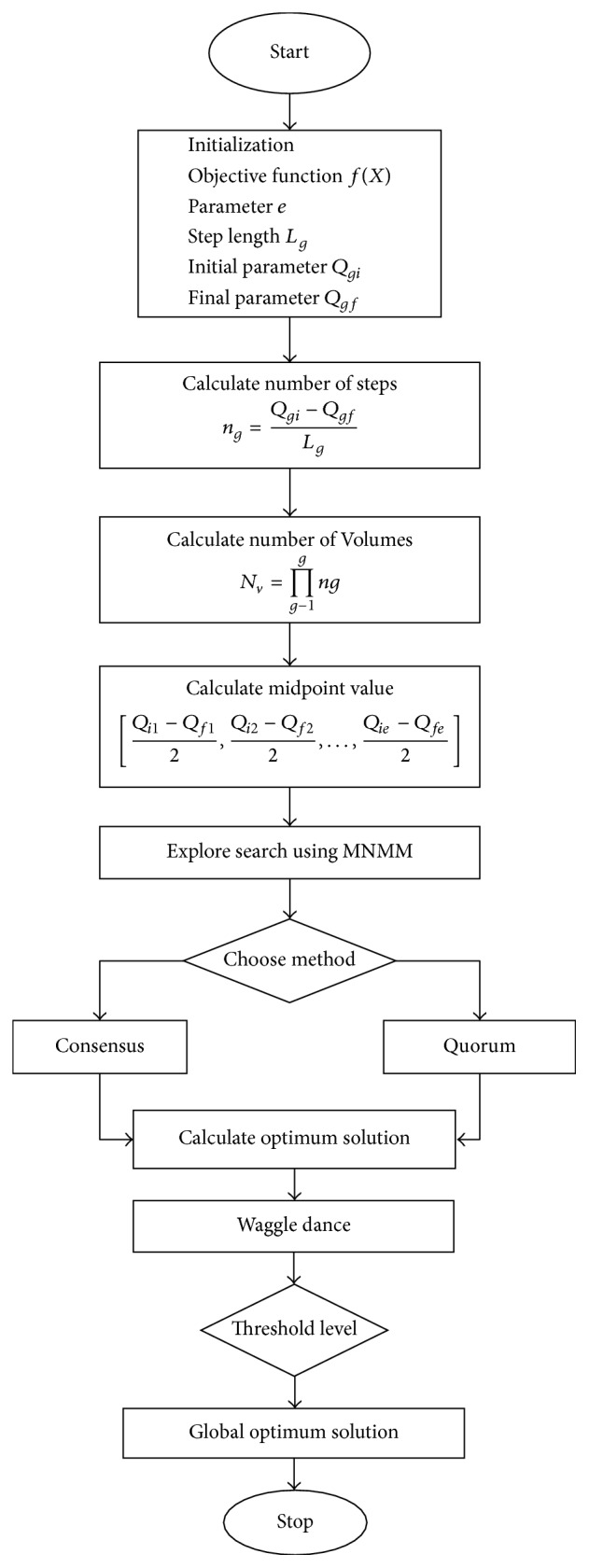
Flowchart of MODBCO.

A key role of the honey bees is to select the best nest site and is done by the process of decision-making to produce a unified decision. They follow a distributed decision-making process to find out the neighbor nest site for their food particles. The pseudocode for the proposed MODBCO algorithm is shown in Algorithm 3.  Figure 6 explains the workflow of the proposed algorithm for the search of food particles by honey bees using MODBCO.

#### 5.2.1. Waggle Dance

The scout bees after returning from the search of food particle report about the quality of the food site by communication mode called waggle dance. Scout bees perform the waggle dance to other quiescent bees to advertise their best nest site for the exploration of food source. In the multiagent system, each agent after collecting individual solution gives it to the centralized systems. To select the best optimal solution for minimal optimal cases, the mathematical formulation can be stated as

(32) $$\begin{array}{c} {dance}_{i}=\min\left(\;f_{i}\left(\;V\;\right)\;\right). \end{array}$$

This mathematical formulation will find the minimal optimal cases among the search solution, where *f*_*i*_(*V*) is the search value calculated by the agent. The search values are recorded in the vector table *V*; *V* is the vector which consists of *e* number of elements. The element *e* contains the value of the parameter; both optimal solution and parameter values are recorded in the vector table.

#### 5.2.2. Consensus

The consensus is the widespread agreement among the group based on voting; the voting pattern of the scout bees is monitored periodically to know whether it reached an agreement and started acting on the decision pattern. Honey bees use the consensus method to select the best search value; the globally optimized point is chosen by comparing the values in the vector table. The globally optimized points are selected using the mathematical formulation

(33) $$\begin{array}{c} f\left(\;V_{g}\;\right)=\min\left[\;f\left(\;V_{1}\;\right),f\left(\;V_{2}\;\right),\ldots ,f\left(\;V_{N_{v}}\;\right)\;\right]. \end{array}$$

#### 5.2.3. Quorum

In quorum method, the optimum solution is calculated as the final solution based on the threshold level obtained by the group decision-making process. When the solution reaches the optimal threshold level *ξ*_*q*_, then the solution is considered as a final solution based on unison decision process. The quorum threshold value describes the quality of the food particle result. When the threshold value is less the computation time decreases, but it leads to inaccurate experimental results. The threshold value should be chosen to attain less computational time with an accurate experimental result.

## 6. Experimental Design and Analysis

### 6.1. Performance Metrics

The performance of the proposed algorithm MODBCO is assessed by comparing with five different competitor methods. Here six performance metrics are considered to investigate the significance and evaluate the experimental results. The metrics are listed in this section.

#### 6.1.1. Least Error Rate

Least Error Rate (LER) is the percentage of the difference between known optimal value and the best value obtained. The LER can be calculated using

(34) $$\begin{array}{c} LER\left(\;\%\;\right)=\overset{r}{\underset{i=1}{\sum }}\frac{{Optimal}_{NRP\text{-}Instance}-{fitness}_{i}}{{Optimal}_{NRP\text{-}Instance}}. \end{array}$$

#### 6.1.2. Average Convergence

The Average Convergence is the measure to evaluate the quality of the generated population on average. The Average Convergence (AC) is the percentage of the average of the convergence rate of solutions. The performance of the convergence time is increased by the Average Convergence to explore more solutions in the population. The Average Convergence is calculated using

(35) AC⁡=∑i=1r1−Avg_fitnessi−OptimalNRP-InstanceOptimalNRP-Instance∗100,

where (*r*) is the number of instances in the given dataset.

#### 6.1.3. Standard Deviation

Standard deviation (SD) is the measure of dispersion of a set of values from its mean value. Average Standard Deviation is the average of the standard deviation of all instances taken from the dataset. The Average Standard Deviation (ASD) can be calculated using

(36) $$\begin{array}{c} ASD=\sqrt{\overset{r}{\underset{i=1}{\sum }}{\left(\text{value obtained in each }{\text{instance}}_{i}-\text{Mean value of the instance}\right)}^{2}}, \end{array}$$

where (*r*) is the number of instances in the given dataset.

#### 6.1.4. Convergence Diversity

The Convergence Diversity (CD) is the difference between best convergence rate and worst convergence rate generated in the population. The Convergence Diversity can be calculated using

(37) $$\begin{array}{c} CD={Convergence}_{best}-{Convergence}_{worst}, \end{array}$$

where Convergence_best_ is the convergence rate of best fitness individual and Convergence_worst_ is the convergence rate of worst fitness individual in the population.

#### 6.1.5. Cost Diversion

Cost reduction is the difference between known cost in the NRP Instances and the cost obtained from our approach. Average Cost Diversion (ACD) is the average of cost diversion to the total number of instances taken from the dataset. The value of ACR can be calculated from

(38) $$\begin{array}{c} ACR=\overset{r}{\underset{i=1}{\sum }}\frac{{Cost}_{i}-{Cost}_{NRP\text{-}Instance}}{\text{Total number of instances}}, \end{array}$$

where (*r*) is the number of instances in the given dataset.

### 6.2. Experimental Environment Setup

The proposed Directed Bee Colony algorithm with the Modified Nelder-Mead Method to solve the NRP is illustrated briefly in this section. The main objective of the proposed algorithm is to satisfy multiobjective of the NRP as follows:

- Minimize the total cost of the rostering problem.
- Satisfy all the hard constraints described in Table 1.
- Satisfy as many soft constraints described in Table 2.
- Enhance the resource utilization.
- Equally distribute workload among the nurses.

The Nurse Rostering Problem datasets are taken from the First International Rostering Competition (INRC2010) by PATAT-2010, a leading conference in Automated Timetabling [38]. The INRC2010 dataset is divided based on its complexity and size into three tracks, namely, sprint, medium, and long datasets. Each track is divided into four types as early, late, hidden, and hint with reference to the competition INRC2010. The first track sprint is the easiest and consists of 10 nurses, 33 datasets which are sorted as 10 early types, 10 late types, 10 hidden types, and 3 hint type datasets. The scheduling period is for 28 days with 3 to 4 contract types, 3 to 4 daily shifts, and one skill specification. The second track is a medium which is more complex than sprint track, and it consists of 30 to 31 nurses, 18 datasets which are sorted as 5 early types, 5 long types, 5 hidden types, and 3 hint types. The scheduling period is for 28 days with 3 to 4 contract types, 4 to 5 daily shifts, and 1 to 2 skill specifications. The most complicated track is long with 49 to 40 nurses and consists of 18 datasets which are sorted as 5 early types, 5 long types, 5 hidden types, and 3 hint types. The scheduling period for this track is 28 days with 3 to 4 contract types, 5 daily shifts, and 2 skill specifications. The detailed description of the datasets available in the INRC2010 is shown in Table 3. The datasets are classified into twelve cases based on the size of the instances and listed in Table 4.

Table 3 describes the detailed description of the datasets; columns one to three are used to index the dataset to track, type, and instance. Columns four to seven will explain the number of available nurses, skill specifications, daily shift types, and contracts. Column eight explains the number of unwanted shift patterns in the roster. The nurse preferences are managed by shift off and day off in columns nine and ten. The number of weekend days is shown in column eleven. The last column indicates the scheduling period. The symbol “*x*” shows there is no shift off and day off with the corresponding datasets.

Table 4 shows the list of datasets used in the experiment, and it is classified based on its size. The datasets present in case 1 to case 4 are smaller in size, case 5 to case 8 are considered to be medium in size, and the larger sized dataset is classified from case 9 to case 12.

The performance of MODBCO for NRP is evaluated using INRC2010 dataset. The experiments are done on different optimization algorithms under similar environment conditions to assess the performance. The proposed algorithm to solve the NRP is coded using MATLAB 2012 platform under Windows on an Intel 2 GHz Core 2 quad processor with 2 GB of RAM. Table 3 describes the instances considered by MODBCO to solve the NRP. The empirical evaluations will set the parameters of the proposed system. Appropriate parameter values are determined based on the preliminary experiments. The list of competitor methods chosen to evaluate the performance of the proposed algorithm is shown in Table 5. The heuristic parameter and the corresponding values are represented in Table 6.

### 6.3. Statistical Analysis

Statistical analysis plays a major role in demonstrating the performance of the proposed algorithm over existing algorithms. Various statistical tests and measures to validate the performance of the algorithm are reviewed by Demšar [39]. The authors used statistical tests like ANOVA, Dunnett test, and post hoc test to substantiate the effectiveness of the proposed algorithm and help to differentiate from existing algorithms.

#### 6.3.1. ANOVA Test

To validate the performance of the proposed algorithm, ANOVA (Analysis of Variance) is used as the statistical analysis tool to demonstrate whether one or more solutions significantly vary [40]. The authors used one-way ANOVA test [41] to show significance in proposed algorithm. One-way ANOVA is used to validate and compare differences between various algorithms. The ANOVA test is performed with 95% confidence interval, the significant level of 0.05. In ANOVA test, the null hypothesis is tested to show the difference in the performance of the algorithms. If the obtained significance value is less than the critical value (0.05), then the null hypothesis is rejected, and thus the alternate hypothesis is accepted. Otherwise, the null hypothesis is accepted by rejecting the alternate hypothesis.

#### 6.3.2. Duncan's Multiple Range Test

After the null hypothesis is rejected, to explore the group differences post hoc or multiple comparison test is performed. Duncan developed a procedure to test and compare all pairs in multiple ranges [42]. Duncan's multiple range test (DMRT) classifies the significant and nonsignificant difference between any two methods. This method ranks in terms of mean values in increasing or decreasing order and group method which is not significant.

### 6.4. Experimental and Result Analysis

In this section, the effectiveness of the proposed algorithm MODBCO is compared with other optimization algorithms to solve the NRP using INRC2010 datasets under similar environmental setup, using performance metrics as discussed. To compare the results produced by MODBCO seems to be more competitive with previous methods. The performance of MODBCO is comparable with previous methods listed in Tables 7–18. The computational analysis on the performance metrics is as follows.

#### 6.4.1. Best Value

The results obtained by MODBCO with competitive methods are shown in Table 7. The performance is compared with previous methods; the number in the table refers to the best solution obtained using the corresponding algorithm. The objective of NRP is the minimization of cost; the lowest values are the best solution attained. In the evaluation of the performance of the algorithm, the authors have considered 69 datasets with diverse size. It is apparently shown that MODBCO accomplished 34 best results out of 69 instances.

The statistical analysis tests ANOVA and DMRT for best values are shown in Table 8. It is perceived that the significance values are less than 0.05, which shows the null hypothesis is rejected. The significant difference between various optimization algorithms is observed. The DMRT test shows the homogenous group; two homogeneous groups for best values are formed among competitor algorithms.

#### 6.4.2. Error Rate

The evaluation based on the error rate shows that our proposed MODBCO yield lesser error rate compared to other competitor techniques. The computational analysis based on error rate (%) is shown in Table 9 and out of 33 instances in sprint type, 18 instances have achieved zero error rate. For sprint type dataset, 88% of instances have attained a lesser error rate. For medium and larger sized datasets, the obtained error rate is 62% and 44%, respectively. A negative value in the column indicates corresponding instances have attained lesser optimum valve than specified in the INRC2010.

**Figure 7:**
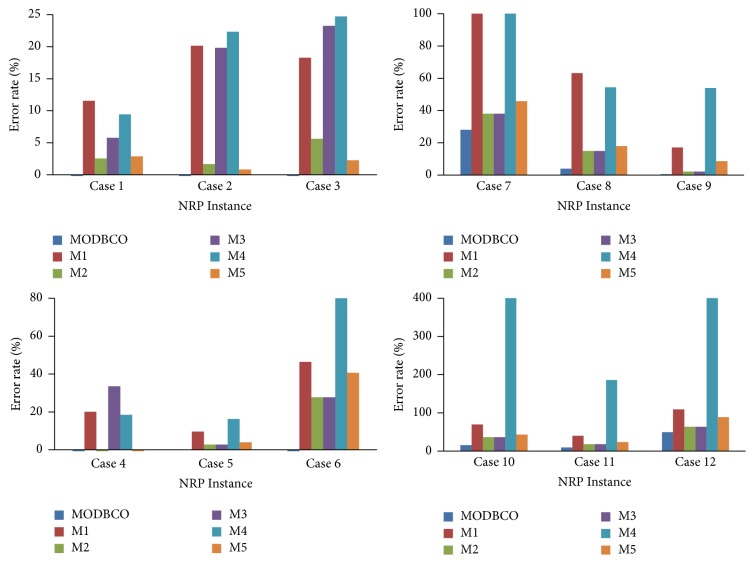
Performance analysis with respect to error rate.

The Competitors M2 and M5 generated better solutions at the initial stage; as the size of the dataset increases they could not be able to find the optimal solution and get trapped in local optima. The error rate (%) obtained by using MODBCO with different algorithms is shown in Figure 7.

The statistical analysis on error rate is presented in Table 10. In ANOVA test, the significance value is 0.000 which is less than 0.05, showing rejection of the null hypothesis. Thus, there is a significant difference in value with respect to various optimization algorithms. The DMRT test indicates two homogeneous groups formed from different optimization algorithms with respect to the error rate.

#### 6.4.3. Average Convergence

**Figure 8:**
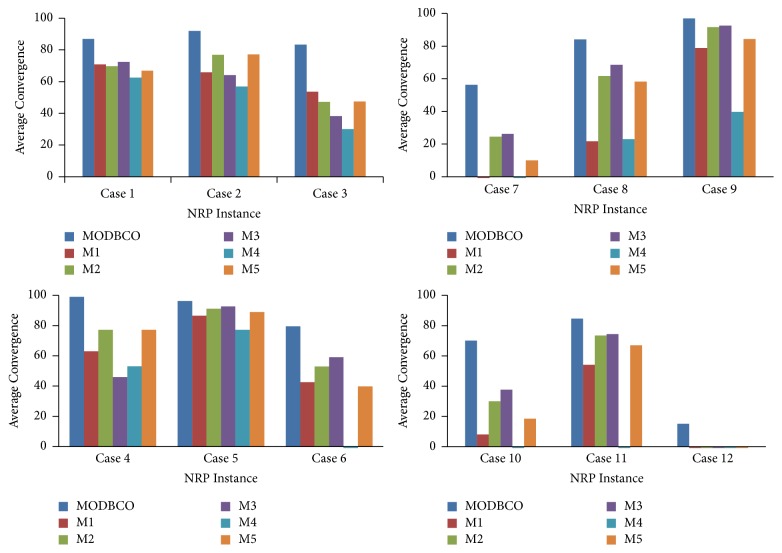
Performance analysis with respect to Average Convergence.

The Average Convergence of the solution is the average fitness of the population to the fitness of the optimal solution. The computational results with respect to Average Convergence are shown in Table 11. MODBCO shows 90% convergence rate in small size instances and 82% convergence rate in medium size instances. For longer instances, it shows 77% convergence rate. Negative values in the column show the corresponding instances get deviated from optimal solution and trapped in local optima. It is observed that with increase in the problem size convergence rate reduces and becomes worse in many algorithms for larger instances as shown in Table 11. The Average Convergence rate attained by various optimization algorithms is depicted in Figure 8.

The statistical test result for Average Convergence is observed in Table 12 with different optimization algorithms. From the table, it is clear that there is a significant difference in mean values of convergence in different optimization algorithms. The ANOVA test depicts the rejection of the null hypothesis since the value of significance is 0.000. The post hoc analysis test shows there are two homogenous groups among different optimization algorithms with respect to the mean values of convergence.

#### 6.4.4. Average Standard Deviation

**Figure 9:**
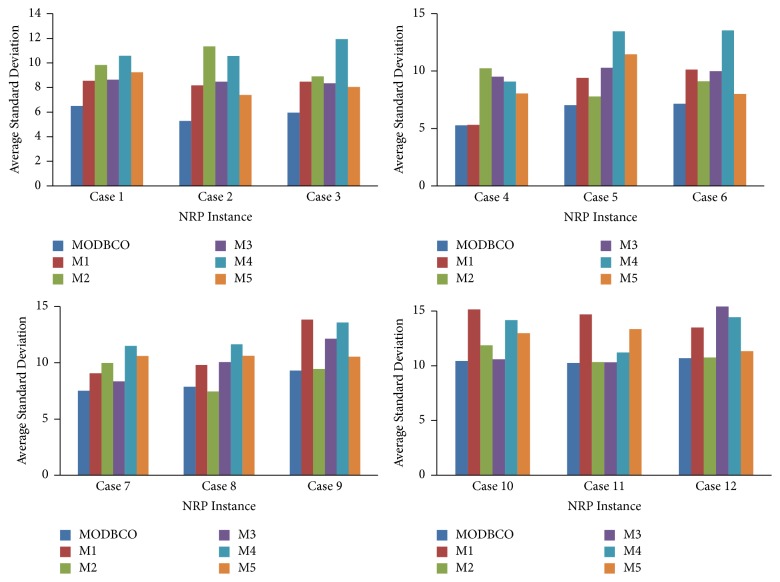
Performance analysis with respect to Average Standard Deviation.

The Average Standard Deviation is the dispersion of values from its mean value, and it helps to deduce features of the proposed algorithm. The computed result with respect to the Average Standard Deviation is shown in Table 13. The Average Standard Deviation attained by various optimization algorithms is depicted in Figure 9.

The statistical test result for Average Standard Deviation is shown in Table 14 with different types of optimization algorithms. There is a significant difference in mean values of standard deviation in different optimization algorithms. The ANOVA test proves the null hypothesis is rejected since the value of significance is 0.00 which is less than the critical value 0.05. In DMRT test, there are three homogenous groups among different optimization algorithms with respect to the mean values of standard deviation.

#### 6.4.5. Convergence Diversity

**Figure 10:**
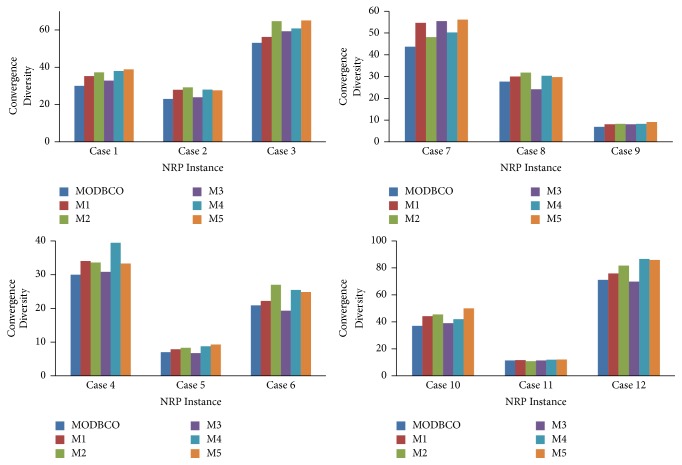
Performance analysis with respect to Convergence Diversity.

The Convergence Diversity of the solution is to calculate the difference between best convergence and worst convergence generated in the population. The Convergence Diversity and error rate help to infer the performance of the proposed algorithm. The computational analysis based on Convergence Diversity for MODBCO with another competitor algorithm is shown in Table 15. The Convergence Diversity for smaller and medium datasets is 58% and 50%. For larger datasets, the Convergence Diversity is 62% to yield an optimum value.  Figure 10 shows the comparison of various optimization algorithms with respect to Convergence Diversity.

The statistical test of ANOVA and DMRT is observed in Table 16 with respect to Convergence Diversity. There is a significant difference in the mean values of the Convergence Diversity with various optimization algorithms. For post hoc analysis test, the significance value is 0.000 which is less than the critical value. Thus the null hypothesis is rejected. From DMRT test, the grouping of various algorithms based on mean value is shown; there are three homogenous groups among the various optimization algorithms with respect to the mean values of the cost diversity.

#### 6.4.6. Average Cost Diversion

**Figure 11:**
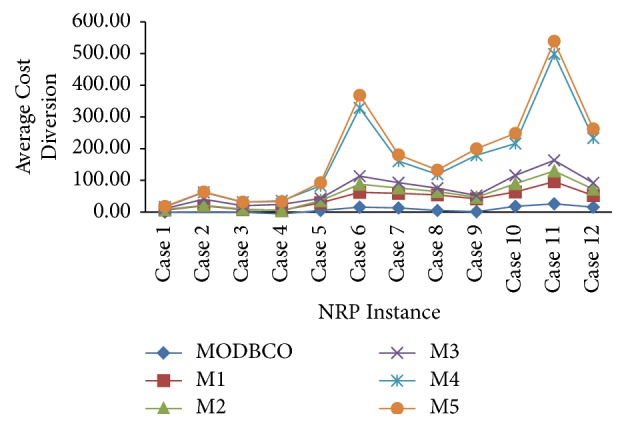
Performance analysis with respect to Average Cost Diversion.

The computational analysis based on cost diversion shows proposed MODBCO yields less diversion in cost compared to other competitor techniques. The computational analysis with respect to Average Cost Diversion is shown in Table 17. For smaller and medium dataset 13% and 38% of instances got diverged out of which many instances yield optimum value. The larger dataset got 56% of cost divergence. A negative value in the table indicates corresponding instances have achieved new optimized values.  Figure 11 depicts the comparison of various optimization algorithms with respect to Average Cost Diversion.

The statistical test of ANOVA and DMRT is observed in Table 18 with respect to Average Cost Diversion. From the table, it is inferred that there is a significant difference in the mean values of the cost diversion with various optimization algorithms. The significance value is 0.000 which is less than the critical value. Thus the null hypothesis is rejected. The DMRT test reveals there are two homogenous groups among the various optimization algorithms with respect to the mean values of the cost diversion.

## 7. Discussion

The experiments to solve NP-hard combinatorial Nurse Rostering Problem are conducted by our proposed algorithm MODBCO. Various existing algorithms are chosen to solve the NRP and compared with the proposed MODBCO algorithm. The results of our proposed algorithm are compared with other competitor methods, and the best values are tabulated in Table 6. To evaluate the performance of the proposed algorithm, various performance metrics are considered to evaluate the efficiency of the MODBCO. Tables 7–18 show the outcome of our proposed algorithm and other existing methods performance. From Tables 7–18 and Figures 7–11, it is evidently shown that MODBCO has more ability to attain the best value on performance metrics compared to competitor algorithms which use the INRC2010.

Compared with other existing methods, the mean value of MODBCO is 19% reduced towards optimum value with other competitor methods, and it attained lesser worst value in addition to the best solution. The datasets are divided based on their size as smaller, medium, and large dataset; the standard deviation of MODBCO is reduced to 4.9%, 2.22%, and 4.13%, respectively. The error rate of our proposed approach, when compared with other competitor methods with various sized datasets, reduces to 10.6% for the smaller dataset, 9.45% for the medium datasets, and 7.04% for the larger datasets. The convergence rate of MODBCO has achieved 90% for the smaller dataset, 82% for the medium dataset, and 77.37% for the larger dataset. The error rate of our proposed algorithm is reduced by 77% when compared with other competitor methods.

The proposed system is tested on larger sized datasets, and it is working astoundingly better than the other techniques. Incorporation of Modified Nelder-Mead in Directed Bee Colony Optimization Algorithm increases the exploitation strategy within the given exploration search space. This method balances the exploration and exploitation without any biased nature. Thus MODBCO converges the population towards an optimal solution at the end of each iteration. Both computational and statistical analyses show the significant performance over other competitor algorithms in solving the NRP. The computational complexity is greater due to the use of local heuristic Nelder-Mead Method. However, the proposed algorithm is better than exact methods and other heuristic approaches in solving the NRP in terms of time complexity.

## 8. Conclusion

This paper tackles solving the NRP using Multiobjective Directed Bee Colony Optimization Algorithm named MODBCO. To solve the NRP effectively Directed Bee Colony algorithm is chosen for global search and Modified Nelder-Mead Method for local best search. The proposed algorithm is evaluated using the INRC2010 dataset, and the performance of the proposed algorithm is compared with other five existing methods. To assess the performance of our proposed algorithm, 69 different cases of various sized datasets are chosen, and 34 out of 69 instances got the best result. Thus, our algorithm contributes with a new deterministic search and effective heuristic approach to solve the NRP. Thus MODBCO outperforms with classical Bee Colony Optimization for solving NRP by satisfying both hard and soft constraints.

The future work can be projected to

- adapting proposed MODBCO for various scheduling and timetabling problems,
- exploring unfeasible solution to imitate optimal solution,
- further tuning the parameters of the proposed algorithm and measuring the exploitation and exploration strategy,
- investigating for applying Second International INRC 2014 datasets.

## Figures and Tables

**Algorithm 1:**
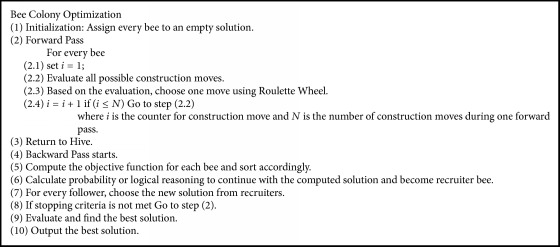
Pseudocode of BCO.

**Algorithm 2:**
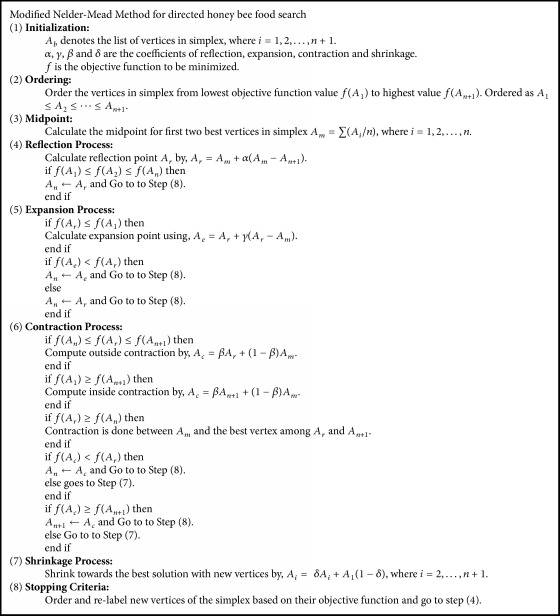
Pseudocode of Modified Nelder-Mead Method.

**Algorithm 3:**
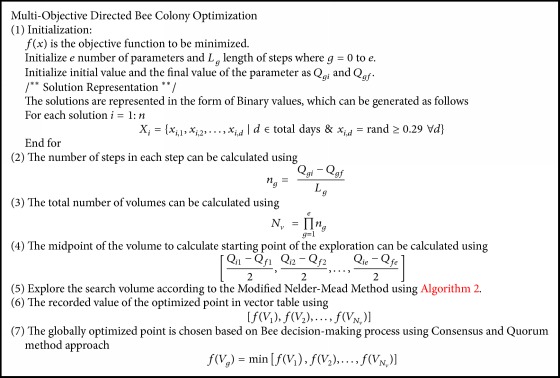
Pseudocode of MODBCO.

**Table 1:** 

Hard constraints	
HC1	All demanded shifts assigned to a nurse.
HC2	A nurse can work with only a single shift per day.
HC3	The minimum number of nurses required for the shift.
HC4	The total number of working days for the nurse should be between the maximum and minimum range.
HC5	A day shift followed by night shift is not allowed.

**Table 2:** 

Soft constraints	
SC1	The maximum number of shifts assigned to each nurse.
SC2	The minimum number of shifts assigned to each nurse.
SC3	The maximum number of consecutive working days assigned to each nurse.
SC4	The minimum number of consecutive working days assigned to each nurse.
SC5	The maximum number of consecutive working days assigned to each nurse on which no shift is allotted.
SC6	The minimum number of consecutive working days assigned to each nurse on which no shift is allotted.
SC7	The maximum number of consecutive working weekends with at least one shift assigned to each nurse.
SC8	The minimum number of consecutive working weekends with at least one shift assigned to each nurse.
SC9	The maximum number of weekends with at least one shift assigned to each nurse.
SC10	Specific working day.
SC11	Requested day off.
SC12	Specific shift on.
SC13	Specific shift off.
SC14	Nurse not working on the unwanted pattern.

**Table 3:** The features of the INRC2010 datasets.

Track	Type	Instance	Nurses	Skills	Shifts	Contracts	Unwanted pattern	Shift off	Day off	Weekend	Time period
Sprint	Early	01–10	10	1	4	4	3	✓	*✓*	2	1-01-2010 to 28-01-2010
	Hidden	01-02	10	1	3	3	4	✓	✓	2	1-06-2010 to 28-06-2010
		03, 05, 08	10	1	4	3	8	✓	✓	2	1-06-2010 to 28-06-2010
		04, 09	10	1	4	3	8	✓	✓	2	1-06-2010 to 28-06-2010
		06, 07	10	1	3	3	4	✓	✓	2	1-01-2010 to 28-01-2010
		10	10	1	4	3	8	✓	✓	2	1-01-2010 to 28-01-2010
	Late	01, 03–05	10	1	4	3	8	✓	✓	2	1-01-2010 to 28-01-2010
		02	10	1	3	3	4	✓	✓	2	1-01-2010 to 28-01-2010
		06, 07, 10	10	1	4	3	0	✓	✓	2	1-01-2010 to 28-01-2010
		08	10	1	4	3	0	×	×	2	1-01-2010 to 28-01-2010
		09	10	1	4	3	0	×	×	2, 3	1-01-2010 to 28-01-2010
	Hint	01, 03	10	1	4	3	8	✓	✓	2	1-01-2010 to 28-01-2010
		02	10	1	4	3	0	✓	✓	2	1-01-2010 to 28-01-2010

Medium	Early	01–05	31	1	4	4	0	✓	✓	2	1-01-2010 to 28-01-2010
	Hidden	01–04	30	2	5	4	9	×	×	2	1-06-2010 to 28-06-2010
		05	30	2	5	4	9	×	×	2	1-06-2010 to 28-06-2010
	Late	01	30	1	4	4	7	✓	✓	2	1-01-2010 to 28-01-2010
		02, 04	30	1	4	3	7	✓	✓	2	1-01-2010 to 28-01-2010
		03	30	1	4	4	0	✓	*✓*	2	1-01-2010 to 28-01-2010
		05	30	2	5	4	7	✓	✓	2	1-01-2010 to 28-01-2010
	Hint	01, 03	30	1	4	4	7	✓	✓	2	1-01-2010 to 28-01-2010
		02	30	1	4	4	7	✓	✓	2	1-01-2010 to 28-01-2010

Long	Early	01–05	49	2	5	3	3	✓	✓	2	1-01-2010 to 28-01-2010
	Hidden	01–04	50	2	5	3	9	×	×	2, 3	1-06-2010 to 28-06-2010
		05	50	2	5	3	9	×	×	2, 3	1-06-2010 to 28-06-2010
	Late	01, 03, 05	50	2	5	3	9	×	×	2, 3	1-01-2010 to 28-01-2010
		02, 04	50	2	5	4	9	×	×	2, 3	1-01-2010 to 28-01-2010
	Hint	01	50	2	5	3	9	×	×	2, 3	1-01-2010 to 28-01-2010
		02, 03	50	2	5	3	7	×	×	2	1-01-2010 to 28-01-2010

**Table 4:** Classification of INRC2010 datasets based on the size.

SI number	Case	Track	Type
1	Case 1	Sprint	Early
2	Case 2	Sprint	Hidden
3	Case 3	Sprint	Late
4	Case 4	Sprint	Hint
5	Case 5	Middle	Early
6	Case 6	Middle	Hidden
7	Case 7	Middle	Late
8	Case 8	Middle	Hint
9	Case 9	Long	Early
10	Case 10	Long	Hidden
11	Case 11	Long	Late
12	Case 12	Long	Hint

**Table 5:** List of competitors methods to compare.

Type	Method	Reference
M_1_	Artificial Bee Colony Algorithm	[14]
M_2_	Hybrid Artificial Bee Colony Algorithm	[15]
M_3_	Global best harmony search	[16]
M_4_	Harmony Search with Hill Climbing	[17]
M_5_	Integer Programming Technique for NRP	[18]

**Table 6:** Configuration parameter for experimental evaluation.

Type	Method
Number of bees	100
Maximum iterations	1000
Initialization technique	Binary
Heuristic	Modified Nelder-Mead Method
Termination condition	Maximum iterations
Run	20
Reflection coefficient	*α* > 0
Expansion coefficient	*γ* > 1
Contraction coefficient	0 > *β* > 1
Shrinkage coefficient	0 < *δ* < 1

**Table 7:** Experimental result with respect to best value.

Instances	Optimal value	MODBCO		M1		M2		M3		M4		M5	
		Best	Worst	Best	Worst	Best	Worst	Best	Worst	Best	Worst	Best	Worst
Sprint early 01	56	56	75	63	74	57	81	59	75	58	77	58	77
Sprint early 02	58	59	77	66	89	59	80	61	82	64	88	60	85
Sprint early 03	51	51	68	60	83	52	75	54	70	59	84	53	67
Sprint early 04	59	59	77	68	86	60	76	63	80	67	84	61	85
Sprint early 05	58	58	74	65	86	59	77	60	79	63	86	60	84
Sprint early 06	54	53	69	59	81	55	79	57	73	58	75	56	77
Sprint early 07	56	56	75	62	81	58	79	60	75	61	85	57	78
Sprint early 08	56	56	76	59	79	58	79	59	80	58	82	57	78
Sprint early 09	55	55	82	59	79	57	76	59	80	61	78	56	80
Sprint early 10	52	52	67	58	76	54	73	55	75	58	78	53	76
Sprint hidden 01	32	32	48	45	67	34	57	43	60	46	65	33	55
Sprint hidden 02	32	32	51	41	59	34	55	37	53	44	68	33	51
Sprint hidden 03	62	62	79	74	92	63	86	71	90	78	96	62	84
Sprint hidden 04	66	66	81	79	102	67	91	80	96	78	100	66	91
Sprint hidden 05	59	58	73	68	90	60	79	63	84	69	88	59	77
Sprint hidden 06	134	128	146	164	187	131	145	203	219	169	188	132	150
Sprint hidden 07	153	154	172	181	204	154	175	197	215	187	210	155	174
Sprint hidden 08	204	201	219	246	265	205	225	267	286	240	260	206	224
Sprint hidden 09	338	337	353	372	390	339	363	274	291	372	389	340	365
Sprint hidden 10	306	306	324	328	347	307	330	347	368	322	342	308	324
Sprint late 01	37	37	53	50	68	38	57	46	66	52	72	39	57
Sprint late 02	42	41	61	53	74	43	61	50	71	56	80	44	67
Sprint late 03	48	45	65	57	80	50	73	57	76	60	77	49	72
Sprint late 04	75	71	87	88	108	75	95	106	127	95	115	74	99
Sprint late 05	44	46	66	52	65	46	68	53	74	57	74	46	70
Sprint late 06	42	42	60	46	65	44	68	45	64	52	70	43	62
Sprint late 07	42	44	63	51	69	46	69	62	81	55	78	44	68
Sprint late 08	17	17	36	17	37	19	41	19	39	19	40	17	39
Sprint late 09	17	17	33	18	37	19	42	19	40	17	40	17	40
Sprint late 10	43	43	59	56	74	45	67	56	74	54	71	43	62
Sprint hint 01	78	73	92	85	103	77	91	103	119	90	108	77	99
Sprint hint 02	47	43	59	57	76	48	68	61	80	56	81	45	68
Sprint hint 03	57	49	67	74	96	52	75	79	97	69	93	53	66
Medium early 01	240	245	263	262	284	247	267	247	262	280	305	250	269
Medium early 02	240	243	262	263	280	247	270	247	263	281	301	250	273
Medium early 03	236	239	256	261	281	244	264	244	262	287	311	245	269
Medium early 04	237	245	262	259	278	242	261	242	258	278	297	247	272
Medium early 05	303	310	326	331	351	310	332	310	329	330	351	313	338
Medium hidden 01	123	143	159	190	210	157	180	157	177	410	429	192	214
Medium hidden 02	243	230	248	286	306	256	277	256	273	412	430	266	284
Medium hidden 03	37	53	70	66	84	56	78	56	69	182	203	61	81
Medium hidden 04	81	85	104	102	119	95	119	95	114	168	191	100	124
Medium hidden 05	130	182	201	202	224	178	202	178	197	520	545	194	214
Medium late 01	157	176	195	207	227	175	198	175	194	234	257	179	194
Medium late 02	18	30	45	53	76	32	52	32	53	49	67	35	55
Medium late 03	29	35	52	71	90	39	60	39	59	59	78	44	66
Medium late 04	35	42	58	66	83	49	57	49	70	71	89	48	70
Medium late 05	107	129	149	179	199	135	156	135	156	272	293	141	164
Medium hint 01	40	42	62	70	90	49	71	49	65	64	82	50	71
Medium hint 02	84	91	107	142	162	95	116	95	115	133	158	96	115
Medium hint 03	129	135	153	188	209	141	161	141	152	187	208	148	166
Long early 01	197	194	209	241	259	200	222	200	221	339	362	220	243
Long early 02	219	228	245	276	293	232	254	232	253	399	419	253	275
Long early 03	240	240	258	268	291	243	257	243	262	349	366	251	273
Long early 04	303	303	321	336	356	306	324	306	320	411	430	319	342
Long early 05	284	284	300	326	347	287	310	287	308	383	403	301	321
Long hidden 01	346	389	407	444	463	403	422	403	421	4466	4488	422	442
Long hidden 02	90	108	126	132	150	120	139	120	139	1071	1094	128	148
Long hidden 03	38	48	63	61	78	54	72	54	75	163	181	54	79
Long hidden 04	22	27	45	49	71	32	54	32	51	113	132	36	58
Long hidden 05	41	55	71	78	99	59	81	59	70	139	157	60	83
Long late 01	235	249	267	290	310	260	278	260	276	588	606	262	286
Long late 02	229	261	280	295	318	265	278	265	281	577	595	263	281
Long late 03	220	259	275	307	325	264	285	264	285	567	588	272	295
Long late 04	221	257	292	304	323	263	284	263	281	604	627	276	294
Long late 05	83	92	107	142	161	104	122	104	125	329	349	118	138
Long hint 01	31	40	58	53	73	44	67	44	65	126	150	50	72
Long hint 02	17	29	47	40	62	32	55	32	51	122	145	36	61
Long hint 03	53	79	137	117	135	85	104	85	101	278	303	102	123

**Table:** (a) ANOVA test

Source factor: best value					
	Sum of squares	df	Mean square	*F*	Sig.
Between groups	1061949	5	212389.8	3.620681	0.003
Within groups	23933354	408	58660.18		
Total	24995303	413			

**Table:** (b) DMRT test

Duncan test: best value			
Method	*N*	Subset for alpha = 0.05	
		1	2
MODBCO	69	120.2319	
M2	69	124.1304	
M5	69	128.087	
M3	69	129.3478	
M1	69	143.1594	
M4	69		263.5507
Sig.		0.629	1.000

**Table 9:** Experimental result with respect to error rate.

Case	MODBCO	M1	M2	M3	M4	M5
Case 1	−0.01	11.54	2.54	5.77	9.41	2.88
Case 2	−0.73	20.16	1.69	19.81	22.35	0.83
Case 3	−0.47	18.27	5.63	23.24	24.72	2.26
Case 4	−9.65	20.03	−2.64	33.48	18.53	−4.18
Case 5	0.00	9.57	2.73	2.73	16.31	3.93
Case 6	−2.57	46.37	27.71	27.71	220.44	40.62
Case 7	28.00	105.40	37.98	37.98	116.36	45.82
Case 8	3.93	63.26	14.97	14.97	54.43	18.00
Case 9	0.52	17.14	2.15	2.15	54.04	8.61
Case 10	15.55	69.70	36.25	36.25	652.47	43.25
Case 11	9.16	40.08	18.13	18.13	185.92	23.41
Case 12	49.56	109.01	63.52	63.52	449.54	88.50

**Table:** (a) ANOVA test

Source factor: error rate					
	Sum of squares	df	Mean square	*F*	Sig.
Between groups	638680.9	5	127736.1796	15.26182	0.000
Within groups	3414820	408	8369.657384		
Total	4053501	413			

**Table:** (b) DMRT test

Duncan test: error rate			
Method	*N*	Subset for alpha = 0.05	
		1	2
MODBCO	69	5.402238	
M2	69	13.77936	
M5	69	17.31724	
M3	69	20.99903	
M1	69	36.49033	
M4	69		121.1591
Sig.		0.07559	1.000

**Table 11:** Experimental result with respect to Average Convergence.

Case	MODBCO	M1	M2	M3	M4	M5
Case 1	87.01	70.84	69.79	72.40	62.59	66.87
Case 2	91.96	65.88	76.93	64.19	56.88	77.21
Case 3	83.40	53.63	47.23	38.23	30.07	47.44
Case 4	99.02	62.96	77.23	45.94	53.14	77.20
Case 5	96.27	86.49	91.10	92.71	77.29	89.01
Case 6	79.49	42.52	52.87	59.08	−139.09	39.82
Case 7	56.34	−32.73	24.52	26.23	−54.91	9.97
Case 8	84.00	21.72	61.67	68.51	22.95	58.22
Case 9	96.98	78.81	91.68	92.59	39.78	84.36
Case 10	70.16	8.16	29.97	37.66	−584.44	18.54
Case 11	84.58	54.10	73.49	74.40	−94.85	66.95
Case 12	15.13	−46.99	−22.73	−9.46	−411.18	−53.43

**Table:** (a) ANOVA test

Source factor: Average Convergence					
	Sum of squares	df	Mean square	*F*	Sig.
Between groups	712642.475	5	142528.495	15.47047	0.0000
Within groups	3758878.26	408	9212.9369		
Total	4471520.73	413			

**Table:** (b) DMRT test

Duncan test: Average Convergence			
Method	*N*	Subset for alpha = 0.05	
		1	2
M4	69	−47.6945	
M1	69		46.4245
M5	69		53.6879
M3	69		57.6296
M2	69		59.5103
MODBCO	69		81.6997
Sig.		1.00	0.054

**Table 13:** Experimental result with respect to Average Standard Deviation.

Case	MODBCO	M1	M2	M3	M4	M5
Case 1	6.501382	8.551285	9.83255	8.645203	10.57402	9.239032
Case 2	5.275281	8.170864	11.33125	8.472083	10.55361	7.382535
Case 3	5.947749	8.474928	8.898058	8.337811	11.93913	8.033767
Case 4	5.270417	5.310443	10.24612	9.50821	9.092851	8.049586
Case 5	7.035945	9.400025	7.790991	10.28423	13.45243	11.46945
Case 6	7.15779	10.13578	9.109779	9.995431	13.54185	8.020126
Case 7	7.502947	9.069255	9.974609	8.341374	11.50138	10.60612
Case 8	7.861593	9.799198	7.447106	10.05138	11.63378	10.61275
Case 9	9.310626	13.83453	9.437943	12.13188	13.56668	10.54568
Case 10	10.42404	15.14141	11.87235	10.59549	14.15797	12.96181
Case 11	10.25454	14.68924	10.33091	10.30386	11.21071	13.3541
Case 12	10.6846	13.49546	10.75478	15.40791	14.42514	11.3357

**Table:** (a) ANOVA test

Source factor: Average Standard Deviation					
	Sum of squares	df	Mean square	*F*	Sig.
Between groups	697.4407	5	139.4881	13.6322	0.000
Within groups	4174.76	408	10.23226		
Total	4872.201	413			

**Table:** (b) DMRT test

Duncan test: Average Standard Deviation				
Method	*N*	Subset for alpha = 0.05		
		1	2	3
MODBCO	69	7.4743		
M2	69		9.615152	
M3	69		9.677027	
M5	69		9.729477	
M1	69		10.13242	
M4	69			11.9316
Sig.		1.00	0.394	1.00

**Table 15:** Experimental result with respect to Convergence Diversity.

Case	MODBCO	M1	M2	M3	M4	M5
Case 1	30.00	35.25	37.28	32.83	37.94	38.83
Case 2	22.96	27.92	29.18	23.85	27.95	27.62
Case 3	53.05	56.21	64.66	59.29	60.79	65.05
Case 4	29.99	34.03	33.62	30.84	39.46	33.32
Case 5	7.01	7.87	8.33	6.71	8.77	9.29
Case 6	20.89	22.21	26.98	19.29	25.45	24.87
Case 7	43.69	54.66	48.13	55.47	50.24	56.18
Case 8	27.67	30.03	31.83	24.11	30.35	29.69
Case 9	6.89	8.10	8.22	8.04	8.24	9.10
Case 10	37.10	44.29	45.53	38.95	41.91	49.98
Case 11	11.43	11.64	10.81	11.36	11.91	12.15
Case 12	91.13	75.96	81.78	69.90	86.63	85.88

**Table:** (a) ANOVA test

Source factor: Convergence Diversity					
	Sum of squares	df	Mean square	*F*	Sig.
Between groups	514.4758	5	102.8952	9.287168	0.000
Within groups	4520.348	408	11.07928		
Total	5034.824	413			

**Table:** (b) DMRT test

Duncan test: Average Standard Deviation				
Method	*N*	Subset for alpha = 0.05		
		1	2	3
M3	69	18.363232		
MODBCO	69	18.42029		
M1	69		19.68116	
M2	69		20.57971	
M4	69		20.68116	
M5	69			21.2464
Sig.		0.919	0.096	1

**Table 17:** Experimental result with respect to Average Cost Diversion.

Case	MODBCO	M1	M2	M3	M4	M5
Case 1	0.00	6.40	1.40	3.20	5.20	1.60
Case 2	−1.00	21.20	0.80	19.60	21.90	0.80
Case 3	−0.40	8.10	1.80	10.60	11.00	0.90
Case 4	−5.67	11.33	−1.67	20.33	11.00	−2.33
Case 5	5.20	24.00	6.80	6.80	40.00	9.80
Case 6	15.80	46.40	25.60	25.60	215.60	39.80
Case 7	13.20	46.00	16.80	16.80	67.80	20.20
Case 8	5.00	49.00	10.67	10.67	43.67	13.67
Case 9	1.20	40.80	5.00	5.00	127.60	20.20
Case 10	18.00	45.40	26.20	26.20	100.00	32.60
Case 11	26.00	70.00	33.60	33.60	335.40	40.60
Case 12	15.67	36.33	20.00	20.00	141.67	29.00

**Table:** (a) ANOVA test

Source factor: Average Cost Diversion					
	Sum of squares	df	Mean square	*F*	Sig.
Between groups	1061949	5	212389.8	4.919985	0.000
Within groups	17612867	408	43168.79		
Total	18674816	413			

**Table:** (b) DMRT test

Duncan test: Average Cost Diversion			
Method	*N*	Subset for alpha = 0.05	
		1	2
MODBCO	69	6.202899	
M2	69	10.10145	
M5	69	14.05797	
M3	69	15.31884	
M1	69	29.13043	
M4	69		149.5217
Sig.		0.573558	1
